# Waist circumference is an effect modifier of the association between bone mineral density and glucose metabolism

**DOI:** 10.20945/2359-3997000000040

**Published:** 2018-05-07

**Authors:** Lygia N. Barroso, Dayana R. Farias, Marcia Soares-Mota, Heloisa Bettiol, Marco Antônio Barbieri, Milton Cesar Foss, Antônio Augusto M. da Silva, Gilberto Kac

**Affiliations:** 1 Universidade Federal do Rio de Janeiro Universidade Federal do Rio de Janeiro (UFRJ) Instituto de Nutrição Josué de Castro Departamento de Nutrição Social e Aplicada Rio de Janeiro RJ Brasil Observatório de Epidemiologia Nutricional, Departamento de Nutrição Social e Aplicada, Instituto de Nutrição Josué de Castro, Universidade Federal do Rio de Janeiro (UFRJ), Cidade Universitária, Ilha do Fundão, Rio de Janeiro, RJ, Brasil; 2 Universidade Federal do Rio de Janeiro Universidade Federal do Rio de Janeiro (UFRJ) Instituto de Nutrição Josué de Castro Rio de Janeiro RJ Brasil Instituto de Nutrição Josué de Castro, Universidade Federal do Rio de Janeiro (UFRJ), Cidade Universitária, Ilha do Fundão, Rio de Janeiro, RJ, Brasil; 3 Universidade de São Paulo Universidade de São Paulo Faculdade de Medicina de Ribeirão Preto Departamento de Puericultura e Pediatria Ribeirão Preto SP Brasil Departamento de Puericultura e Pediatria, Faculdade de Medicina de Ribeirão Preto, Universidade de São Paulo, Ribeirão Preto, SP, Brasil; 4 Universidade Federal do Maranhão Universidade Federal do Maranhão (UFMA) Centro de Ciências da Saúde Departamento de Saúde Pública São Luís MA Brasil Departamento de Saúde Pública, Centro de Ciências da Saúde, Universidade Federal do Maranhão (UFMA), São Luís, MA, Brasil

**Keywords:** Insulin resistance, glucose metabolism, bone mineral density, bone turnover markers, osteocalcin, bone formation markers

## Abstract

**Objective:**

The role of bone markers on insulin resistance (IR) remains controversial. The objective of this study is to evaluate the association between bone mineral density (BMD) and glucose metabolism and investigate if visceral hyperadiposity, evaluated by waist circumference (WC), is an effect modifier of this association.

**Subjects and methods:**

Cross-sectional analysis with 468 young adults from the fourth follow-up of the 1978/79 Ribeirão Preto prospective birth cohort, Brazil. BMD, total osteocalcin (OC), fasting plasma glucose and insulin concentrations were assessed. IR, sensitivity (S) and secretion (β) were estimated by homeostasis model assessment (HOMA) indexes. Multiple linear regression models were constructed to estimate the association between BMD and glucose metabolism. Beta coefficient, R^2^ and p-values were provided. WC was tested as an effect modifier and OC as a confounder. The covariates were selected based on Direct Acyclic Graph.

**Results:**

Significant interaction between BMD (femoral neck and proximal femur areas) and WC on glucose metabolism was observed in the adjusted models. Subjects with increased WC presented a positive association between BMD and log HOMA1-IR while an inverse association was found in those with normal WC (femoral neck R^2^ = 0.17, p = 0.036; proximal femur R^2^ = 0.16, p = 0.086). BMD was negatively associated with log HOMA2-S in individuals with increased WC and positively in those with normal WC (femoral neck R^2^ = 0.16, p = 0.042; proximal femur R^2^ = 0.15, p = 0.097). No significant associations between BMD, log HOMA2-β and OC and glucose metabolism markers were observed.

**Conclusions:**

BMD was associated with glucose metabolism, independently of OC, and WC modifies this association.

## INTRODUCTION

Insulin resistance (IR) is characterized by a reduction of the action of the hormone in targets sites, such as muscle, adipose tissue and the liver, resulting in hyperglycemia. To maintain glucose homeostasis, the pancreas adapts through changes in the pancreatic β-cells, resulting in increased insulin secretion. However, exceeding the functional and adaptive capacity can result in the development of type 2 diabetes mellitus (DM2) (1).

Several factors, such as being overweight, age, sex, skin color and lifestyle (physical activity, smoking and alcohol intake) (2–8), may be involved in the etiology of IR. Visceral obesity, is associated with a chronic inflammatory response associated with the development of IR (1). In addition to these classic factors, a possible role of bone markers in IR was found in experimental models (9).

Bone mineral density (BMD) results of the remodeling process, i.e., complex process of bone reabsorption and formation, which include the participation of calcitropic hormones that act directly on osteoblasts, osteoclasts and osteocytes. Osteocalcin (OC) is a protein synthesized by osteoblasts during bone formation and therefore affected by the concentration of calcitropic hormones, such as calcitonin and the parathyroid hormone (10). The visceral adipose tissue is associated with the genesis of osteoclasts and therefore with increased bone reabsorption (11). Thus, this tissue can affect bone turnover and the concentration of bone turnover markers, such as OC, which seems to be inversely associated with body fat in Chinese men (12). Recent investigations have studied the effects of bone turnover markers in glucose metabolism, adding evidence of the existence of a possible bone-pancreas endocrine axis (9,13). OC seems to be positively associated with proliferation of pancreatic β-cells, insulin secretion and sensitivity and inversely associated with IR in experimental models. In humans, these associations remains controversial in the literature (9,14,15).

The association between bone and glucose metabolism is not well defined and few studies have sought to study BMD in this context. We expect an inverse association between BMD and IR in young adults without visceral hyperadiposity, which also appear to have a higher concentration of OC (12), and tested if this is dependent of OC. In individuals with increased waist circumference (WC), we suspect that this association can be modified due to changes in bone metabolism. So we considered WC as a modifying effect on the association between bone and glucose metabolism.

The objective of this study was to evaluate if BMD predicts alterations in glucose metabolism, and assess the potential role of OC in this association. In addition, the relationship between OC and WC was tested in our sample.

## SUBJECTS AND METHODS

### Study design and participants

This cross-sectional study was developed with data collected in the fourth phase of the prospective cohort study of individuals born in Ribeirão Preto from the 1^st^ of June 1978 to the 31^st^ of May, 1979. At baseline, information was obtained from 9,067 live newborns delivered in the maternity hospitals of Ribeirão Preto. Infants born to mothers who did not reside in the municipality (n = 2,094) and twins (n = 146) were excluded from the original study. The initial sample comprised 6,827 infants born to mothers residing in Ribeirão Preto.

In 2002, when the fourth cohort follow-up was conducted, 5,665 young adults between 23 and 25 years of age were identified as living in the city. Ribeirão Preto consists of 4 geo-economic regions. A sub sample was created from the original study, of which one of three individuals who lived in the same geo-economic area were invited to participate in this phase of the study, resulting in a total of 2,063 young adults.

Of the 2,063 individuals included in the fourth phase of the cohort, 513 agreed to undergo the BMD evaluation. Seventeen subjects were excluded due to the presence of a condition that would interfere with the clinical assessment or measures of bone metabolism (i.e., type 1 diabetes, asthmatics using corticosteroids, amaurosis, anorexia nervosa, scoliosis, urolithiasis and stroke). Additionally, 28 subjects were lost due to missing data or because their total OC and markers of glucose metabolism (fasting glucose and insulin) were not measured. The final sample of the present study comprised 468 individuals (females = 235) who underwent BMD, OC and HOMA [(homeostasis model assessment (HOMA), IR, insulin sensitivity (S), and β-cell function (β)] evaluations. More detailed information about this cohort can be obtained from previous publication (16).

### Measurements

A 40-mL blood sample was collected after 12-hour fasting period. All laboratory tests (fasting insulin and glucose and OC) were analyzed at the time of data collection. Fasting glucose and insulin were determined using commercial kits by GOD/PAP human diagnostic colorimetric enzymatic method (Chronolab AG, Zug, Switzerland) and radioimmunoassay (Insulin kit, DPC, Los Angeles, CA, USA), respectively. OC was determined using an immunoradiometric method (DSL-7600, IRMA, Webster, TX, USA).

IR was estimated using the original HOMA1-IR, calculated according to the formula: fasting plasma glucose (mmol/L) x fasting plasma insulin (µU/mL)/22.5. To estimate insulin sensitivity (HOMA2-S) and secretion (HOMA2-β), we used the HOMA computer model (HOMA2 model), available from https://www.dtu.ox.ac.uk/homacalculator/.

BMD (g/cm^2^) were obtained by dual-energy X-ray absorptiometry (DXA) using Hologic QDR-4500 (Waltham, MA, USA) equipment. Measurements of absolute precision error (and the percentage coefficient of variation) for BMD were 0.007 g/cm^2^ (0.66%), 0.015 g/cm^2^ (1.77%) and 0.007 g/cm^2^ (0.70%) for the three evaluated anatomic areas: the lumbar spine, femoral neck and total proximal femur, respectively. The phantom coefficient of variation throughout the study was 0.38%. A standardized technician performed the quality control and all measurements. The analysis was performed in the nuclear medicine laboratory of the Clinics Hospital, Faculty of Medicine of Ribeirão Preto, University of São Paulo, Brazil.

The following socio-demographic and lifestyle variables were obtained through structured questionnaires: sex (male; female), age (23–25), self-classified skin color (white; mulatto/black/yellow), schooling (≤ 8; 9-11 and ≥ 12 years of study) and smoking (smokers; non-smokers).

Physical activity was measured using the short version of the International Physical Activity Questionnaire (IPAQ), validated for the Brazilian population, and categorized as low, moderate, or high activity (17).

Caloric intake (kcal/day) was estimated based on an adaptation of a validated food frequency questionnaire (FFQ) (18). The software Dietsys version 4.0 was used (National Cancer Institute, Bethesda, MD, USA). Alcohol consumption was estimated based on the FFQ, expressed as a percentage of total dietary energy per day.

Adult weight and height were obtained using standardized techniques. A mechanical scale (Filizola, São Paulo, Brazil) with an accuracy of 100 g and a freestanding wood stadiometer (University of São Paulo, Ribeirão Preto, Brazil) with an accuracy of 0.1 cm were used. Body mass index (BMI) was categorized as < 25 (underweight or normal weight), 25 to 29.9 (overweight) and ≥ 30 kg/m^2^ (obesity) (19).

Waist circumference (WC) was measured by a D-loop non-stretch fiberglass tape as the smallest circumference between the ribs and the iliac crest while the subject stood with the abdomen relaxed at the end of a normal expiration. The individuals were classified as normal/increased WC (women: < 80 cm; ≥ 80 cm; men: < 94 cm; ≥ 94 cm) according to cutoff points of WC proposed by the World Health Organization (WHO) (20).

### Ethics

This study was approved by the Research Ethics Committee of the Clinics Hospital, Faculty of Medicine of Ribeirão Preto, University of São Paulo, Brazil, in February 2000 (protocol no. 7606/99).

### Statistical analyses

The subject characteristics were described using means (standard deviation) and p-value refers to the Student's *t*-test or median (interquartile range) and Mann-Whitney U test. Categorical variables are expressed as absolute and relative frequency and compared by qui-squared test.

The study outcomes were described using medians and interquartile ranges stratified by categories of potentially associated factors. Continuous variables were categorized into tertiles, and comparisons between categories were performed using Mann-Whitney U and Kruskal-Wallis tests.

To evaluate the association between BMD and IR (HOMA1-IR), sensitivity (HOMA2-S) and secretion (HOMA2-β), multiple linear regression models were fitted for each outcome. For this analysis, HOMA1-IR, HOMA2-S and HOMA2-β were log-transformed.

WC was tested as an effect modifier in multiple linear regression models considering that inflammation associated with visceral adipose tissue can affect bone metabolism markers, which may explain the association between BMD and IR. We constructed linear prediction plots of the associations between each anatomic bone area (spinal, femoral neck and proximal femur) and the outcomes, stratified by WC cutoff points (normal/increased), in order to interpret the interactions.

**Figure S1:**
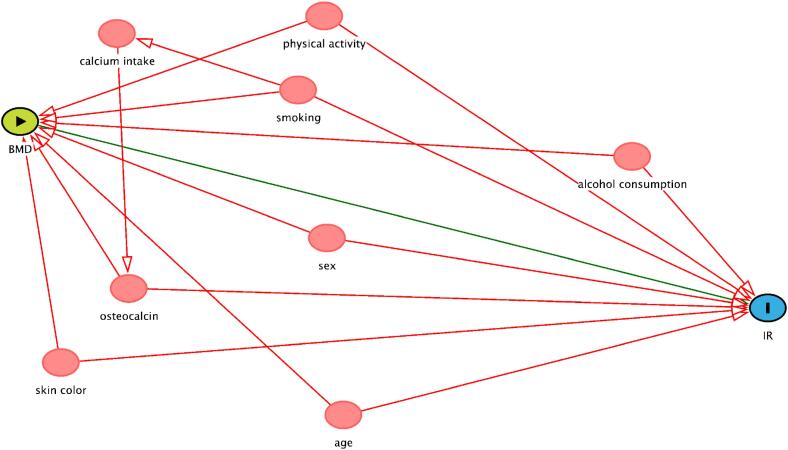
Causal diagram of the association between BMD and IR. Minimal sufficient adjustment sets for estimating the total effect of BMD and IR, suggested by DAG – age, alcohol consumption, osteocalcin, physical activity, sex, skin color and smoking. Colors of variables: green – exposure; blue – outcome; red – co variables.

The covariates were selected for inclusion in the final model based on a directed acyclic graph [(DAG, www.dagitty.net), Figure S1]. A DAG is a graphic model in which potential confounding factors that can distort the causal inference process can be identified and included as covariates in adjusted models (21). DAGs can make more explicit the relationship between exposure and outcome and help avoid inappropriate adjustments.

OC concentration was included in the models as a confounder because of its association with both the exposure and the outcome. We tested whether the association between BMD and glucose metabolism is dependent of OC or if there is an independent pathway linking BMD and IR. The relationship between OC and WC was tested by Pearson correlation test, stratified by sex.

In the analysis, associations with p-value < 0.05 were considered significant, except in the evaluation of interactions, in which a p-value < 0.1 was considered significant (22). The regression analysis provided a beta coefficient, the co-variable for the p-value, the R^2^ (variation explained by the models) for each model, and the p-value of all the multiple models. All analyses were performed using Stata Data Analysis and Statistical Software (STATA) version 12.0, 2011, College Station, TX (StataCorp LP).

## RESULTS

We evaluated 468 (233 men and 235 women) adults. The study participants had a mean age of 23.5 (0.5) years and a mean BMI of 23.7 (4.2) kg/m². The majority of the sample was white (65%), presented normal WC (78%), had more than 8 years of schooling (88%), reported moderate or high physical activity (74%) and were non-smokers (85%). The mean calorie intake and alcohol consumption were 2,188.9 (713.4) kcal/day and 1.8% (2.3) of EI/day, respectively. The median (interquartile range) of IR, insulin sensitivity and β-cell function were 1.1 (0.7:1.7), 136.6 (92.7:217.9) and 98.1 (73.7:132.4), respectively. Men presented higher IR and lower insulin secretion compared to women (p < 0.05). The mean OC concentration was 12.6 (5.1) ng/mL, and the BMD was 1.0 (0.1) g/cm^2^ for the spinal anatomic area, 0.9 (0.2) g/cm^2^ for the femoral neck and 1.0 (0.2) g/cm^2^ for the proximal femur. Higher mean of OC and BMD (spinal, femoral neck and proximal femur) were detected among men (p < 0.001) (Table 1).

**Table 1:** Descriptive characteristics of a young adults sample, 2002-2004 Ribeirão Preto cohort, Brazil, fourth follow-up

Characteristics		Total (n = 468)	Men (n = 233)	Women (n = 235)	*p*-value^1^
Age (years)		23.5 (0.5)	23.5 (0.03)	23.5 (0.03)	0.312
BMI (kg/m^2^)		23.7 (4.2)	24.7 (0.27)	22.7 (0.26)	**< 0.001**
WC (cm)^2^					**0.025**
	Normal	363 (78.0)	180 (49.6)	183 (50.4)	
	Increased	105 (22.0)	53 (50.5)	52 (49.5)	
Skin color					0.070
	White	304 (65.0)	142 (46.7)	162 (53.3)	
	Black/mullato/yellow	164 (35.0)	91 (55.5)	73 (44.5)	
Schooling (years)					0.277
	≤ 8	58 (12.0)	27 (46.5)	31 (53.5)	
	9-11	262 (56.0)	139 (53.0)	123 (47.0)	
	≥ 12	148 (32.0)	67 (45.3)	81 (54.7)	
Energy intake (kcal/day)		2188.9 (713.4)	2415.9 (45.8)	1963.8 (42.6)	**< 0.001**
Alcohol consumption (% of EI/day)		1.8 (2.3)	2.44 (0.16)	1.13 (0.12)	**< 0.001**
Physical activity					**0.002**
	Low	121 (26)	46 (38.0)	75 (62.0)	
	Moderate	200 (43)	99 (49.5)	101 (50.5)	
	High	147 (31)	88 (59.9)	59 (40.1)	
Smoking					**0.016**
	Yes	68 (85.0)	43 (63.2)	25 (36.8)	
	No	400 (15.0)	190 (47.5)	210 (52.5)	
HOMA1-IR		1.1 (0.7;1.7)	1.26 (0.78;1.92)	1.1 (0.7;1.5)	**0.016**
HOMA2-S		136.6 (92.7;217.9)	131.5 (83.1;204.8)	140.2 (100;223.6)	0.104
HOMA2-β		98.1 (73.7;132.4)	92.4 (69.9;131)	104.3 (76.5;134.2)	**0.024**
Osteocalcin (ng/mL)		12.6 (5.1)	14.01 (0.33)	11.22 (0.31)	**< 0.001**
BMD Spinal (g/cm^2^)		1.0 (0.1)	1.06 (0.00)	0.99 (0.00)	**< 0.001**
BMD Femoral neck (g/cm^2^)		0.9 (0.2)	1.00 (0.01)	0.84 (0.00)	**< 0.001**
BMD Proximal femur (g/cm^2^)		1.0 (0.2)	1.09 (0.01)	0.90 (0.00)	**< 0.001**

Continuous variables are expressed as mean (standard deviation)

1 *p*-value refers to the Student's *t*-test or median (interquartile range) and Mann-Whitney U test. Categorical variables are expressed as absolute and relative frequency and compared by qui-squared test.

2 Categorized using World Health Organization cutoff points, normal WC: < 80 cm for women and < 94 cm for men; increased WC: ≥ 80 cm for women and ≥ 94 cm for men.

For EI variable, we had 1 exclusion due to high calorie value (> 6000 kcal/day).

BMI: body mass index; WC: waist circumference; EI: energy intake; HOMA: homeostatic model assessment; HOMA1-IR: insulin resistance; HOMA2-S: insulin sensitivity; HOMA2-β: β-cell function (insulin secretion); BMD: bone mineral density.

A positive association between nutritional status markers (BMI and WC) and HOMA1-IR and HOMA2-β and an inverse association with HOMA2-S was found (p < 0.001 for all). Individuals who reported low physical activity had higher median HOMA1-IR (p = 0.007) and HOMA2-β values (p < 0.001) and lower HOMA2-S values (p = 0.002) than those reporting moderate or high levels of physical activity. The median HOMA1-IR and HOMA2-β levels differed between the sexes, i.e., men presented higher mean IR values (p = 0.016) and lower hormone secretion (p = 0.024) than women. Subjects classified in the 1^st^ tertile of OC presented significantly higher median levels of HOMA2-β than those in the 2^nd^ and 3^rd^ tertiles (p = 0.018). The femoral neck and proximal femur BMD were inversely associated with insulin sensitivity (p = 0.036 and p = 0.002) and were positively associated with IR (p = 0.013 and p < 0.001) (Table 2).

**Table 2:** Distribution of insulin resistance (HOMA1-IR^1^), insulin sensitivity (HOMA2-S^2^) and β cell function (HOMA2-β^2^) in 468 young adults according to categories of selected variables, 2002-2004 Ribeirão Preto cohort, Brazil, fourth follow-up

		n	HOMA1–IR	*p*1	HOMA2-S	*p*1	HOMA2-β	*p*1
BMI (kg/m^2^)								
	< 25	312	1.0 (0.6;1.4)ᵃ		157.9 (110.6;247.2)ᵃ		92.0 (70.2;122.2)ᵃ	
	≥ 25-29.9	116	1.4 (0.9;1.9)^b^		116.3 (84.9;170.1)^b^		108.9 (77.9;139.5)^b^	
	≥ 30	40	2.5 (1.5;3.3)^c^	**< 0.001**	68.9 (50.3;100.0)^c^	**< 0.001**	144.4 (109.8;182.0)^c^	**< 0.001**
WC^2^								
	Normal	363	1.0 (0.6;1.5)		154.5 (108.0;242.7)		92.3 (70.5;122.0)	
	Increased	105	1.8 (1.1;3.2)	**< 0.001**	88.9 (55.4;135.4)	**< 0.001**	129.4 (95.0;165.5)	**< 0.001**
Sex								
	Women	235	1.1 (0.7;1.5)		140.2 (100.0;224.8)		104.3 (76.5;134.2)	
	Men	233	1.3 (0.8-1.9)	**0.016**	131.5 (83.1;204.8)	0.105	92.4 (69.9;131.0)	**0.024**
Physical activity								
	Low	121	1.3 (0.9;2.0)ᵃ		118.5 (80.9;174.7)ᵃ		111.8 (88.5;147.5)ᵃ	
	Moderate	200	1.1 (0.7;1.6)^b^		136.7 (101.4;228.0)^b^		94.6 (72.8;126.3)^b^	
	High	147	1.0 (0.6;1.7)^b^	**0.007**	152.1 (93.9;262.4)^b^	**0.002**	87.5 (67.0;125.8)^b^	**< 0.001**
Smoking								
	No	400	1.1 (0.7;1.7)		136.0 (95.0;217.9)		98.7 (73.7;131.3)	
	Yes	68	1.1 (0.7;1.9)	0.907	146.5 (82.5;214.1)	0.890	94.5 (69.9;147.9)	0.955
Skin color								
	White	304	1.1 (0.7;1.6)		141.1 (97.8;223.9)		95.1 (72.4;129.3)	
	Black/mullato/yellow	164	1.2 (0.8;2.0)	0.055	128.8 (81.2;194.3)	0.055	100.4 (74.9;141.5)	0.154
Schooling (years)								
	≤ 8	58	1.2 (0.7;2.0)		117.6 (77.2;208.6)		104.9 (76.4;135.8)	
	9-11	262	1.2 (0.7;1.7)		133.6 (90.2;211.9)		97.5 (72.9;131.7)	
	≥ 12	148	1.1 (0.7;1.6)	0.177	151.2 (100.7;221.6)	0.117	95.6 (73.8;132.5)	0.349
Alcohol consumption (% of EI/day)								
	1^st^ and 2^nd^ tertiles (0.0-2.1)	315	1.1 (0.7;1.7)		136.1 (93.9;217.5)		98.3 (74.8;134.0)	
	3^rd^ tertile (2.2-12.1)	153	1.1 (0.7;1.7)	0.727	139.8 (89.6;218.4)	0.857	98.0 (69.1;130.2)	0.479
Energy intake (kcal/day)								
	1^st^ and 2^nd^ tertiles (851.1-2377.6)	313	1.1 (0.7;1.6)		141.3 (96.6;211.9)		98.9 (73.8;134.2)	
	3^rd^ tertile (2394.1-4940.7)	154	1.3 (0.7;1.8)	0.221	125.7 (88.5;219.1)	0.363	96.5 (72.1;131.0)	0.602
Osteocalcin (ng/mL)								
	1^st^ tertile (2.9-9.6)	151	1.2 (0.8;1.9)		131.8 (83.5;197.6)		106.6 (78.5;144.6)ᵃ	
	2^nd^ tertile (9.8-14.3)	161	1.1 (0.7;1.6)		139.8 (99.7;219.1)		93.8 (72.9;130.2)^b^	
	3^rd^tertile (14.4-32.9)	156	1.1 (0.6;1.7)	0.174	151.1 (95.0;238.6)	0.110	93.4 (68.6;120.7)^b^	**0.018**
BMD Spinal (g/cm^2^)								
	1^st^ and 2^nd^ tertiles (0.7-1.1)	309	1.1 (0.7;1.6)		141.0 (97.2;224.3)		98.5 (73.1;130.2)	
	3^rd^ tertile (1.1-1.4)	159	1.2 (0.7;1.8)	0.062	123.0 (85.4;200.0)	0.089	97.0 (74.5;134.6)	0.415
BMD Femoral neck (g/cm^2^)								
	1^st^ and 2^nd^ tertiles (0.5-1.0)	310	1.1 (0.7;1.6)		140.6 (99.4;229.5)		99.9 (73.7;130.9)	
	3^rd^ tertile (1.0-1.5)	158	1.3 (0.8;1.9)	**0.013**	125.7 (80.9;197.2)	**0.036**	95.3 (73.7;135.8)	0.864
BMD Proximal femur (g/cm^2^)								
	1^st^ and 2^nd^ tertiles (0.6-1.0)	309	1.1 (0.7;1.5)		147.2 (100.0;229.5)		99.0 (73.7;129.3)	
	3^rd^ tertile (1.0-1.5)	159	1.4 (0.9;2.3)	**< 0.001**	115.7 (75.0;180.8)	**0.002**	96.3 (73.7;141.4)	0.447

1 p-value refers to Kruskall Wallis test and Mann-Whitney U test. Values with differing superscript letters (a, b, c) denote statistically significant differences across the categories.

2 Categorized using World Health Organization cutoff points, normal WC: < 80 cm for women and < 94 cm for men; increased WC: ≥ 80 cm for women and ≥ 94 cm for men.

Data are expressed as median (interquartile range). For EI variable, we had 1 exclusion due to high calorie value (> 6000 kcal/day).

HOMA: homeostatic model assessment; HOMA1-IR: insulin resistance; HOMA2-S: insulin sensitivity; HOMA2-β: β-cell function (insulin secretion); BMI: body mass index; WC: waist circumference; EI: energy intake; BMD: bone mineral density.

A significant inverse correlation between OC and WC was observed in men (r = −0.23, p = 0.002) and women (r = −0.15, p = 0.020) (data not shown).

**Figure 1:**
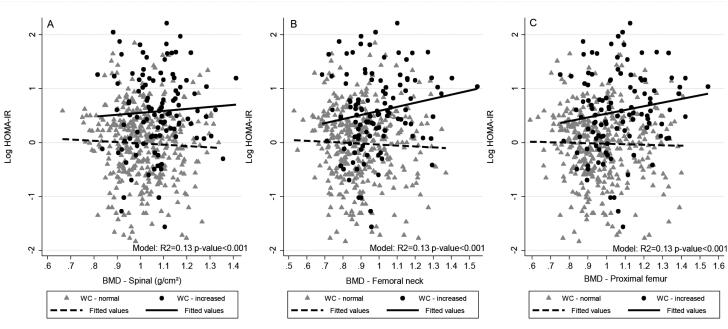
Scatter and linear prediction between BMD and Log HOMA1-IR according to WC in 468 young adults, 2002-2004 Ribeirão Preto, Brazil, fourth cross-sectional evaluation. **A)** Spinal BMD. **B)** Femoral neck BMD. **C)** Proximal femur BMD. HOMA1-IR: homeostatic model assessment – insulin resistance; WC: waist circumference; BMD: bone mineral density. Fitted values were predicted using linear regression models; WC was categorized using World Health Organization cutoff points, normal WC: < 80 cm for women and < 94 cm for men; increased WC: ≥ 80 cm for women and ≥ 94 cm for men.

**Figure 2:**
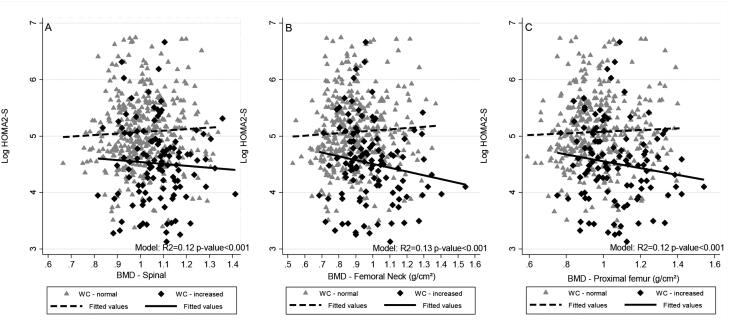
Scatter and linear prediction between BMD and Log HOMA2-S according to WC in 468 young adults, 2002-2004 Ribeirão Preto, Brazil, fourth cross-sectional evaluation. **A)** Spinal BMD. **B)** Femoral neck BMD. **C)** Proximal femur BMD. HOMA2-S: homeostatic model assessment – insulin sensitivity; WC: waist circumference; BMD: bone mineral density. Fitted values were predicted using linear regression models; WC was categorized using World Health Organization cutoff points, normal WC: < 80 cm for women and < 94 cm for men; increased WC: ≥ 80 cm for women and ≥ 94 cm for men.

We found a significant interaction between BMD (femoral neck and proximal femur) and WC in the fully adjusted regression (p < 0.1). We observed a positive association between BMD and the log HOMA1-IR level in individuals with increased WC and an inverse association in those with normal WC (femoral neck R^2^ = 0.17, p=0.036; proximal femur R^2^ = 0.16, p = 0.086). BMD was negatively associated with the log HOMA2-S level in subjects with increased WC and positively associated in those with normal WC (femoral neck R^2^ = 0.16, p = 0.042; proximal femur R^2^ = 0.15, p = 0.097). We did not observe significant associations between BMD (spinal, femoral neck and proximal femur) and the log HOMA2-β level and OC and the log HOMA1-IR, HOMA2-S and HOMA2-β levels (Table 3 and Figures 1 and 2).

**Table 3:** Linear regression between bone mineral density (BMD; g/cm^2^) and insulin resistance (**HOMA1-IR**^1^), insulin sensitivity (**HOMA2-S**^2^) and β cell function (**HOMA2-**β^2^) in 468 young adults, 2002-2004 Ribeirão Preto cohort, Brazil, fourth follow-up

	HOMA1-IR				HOMA2-S				HOMA2-β			
	Model 1 β^1^ (95% CI)	*p*^2^	Model 2 β^1^ (95% CI)	*p*^2^	Model 1 β^1^ (95% CI)	*p*^2^	Model 2 β^1^ (95% CI)	*p*^2^	Model 1 β^1^ (95% CI)	*p*^2^	Model 2 β^1^ (95% CI)	*p*^2^
**BMD - Spinal**												
Spinal (g/cm^2^)	-0.2 (-0.8;0.4)	0.510	-0.5 (-1.1;0.1)	0.133	0.2 (-0.3;0.8)	0.432	0.4 (-0.2;1.0)	0.160	-0.2 (-0.6;0.1)	0.196	-0.2 (-0.5;0.2)	0.424
WC (normal/increased)^3^	-0.0 (-1.4;1.3)	0.936	-0.2 (-1.6;1.2)	0.763	0.1 (-1.2;1.4)	0.885	0.2 (-1.1;1.5)	0.760	-0.1 (-1.0;0.7)	0.735	-0.1 (-1.0;0.8)	0.836
*Interaction term*												
Spinal#WC	0.6 (-0.7;1.9)	0.344	0.8 (-0.5;2.0)	0.246	-0.6 (-1.8;0.6)	0.324	-0.7 (-1.9;0.5)	0.256	0.4 (-0.4;1.2)	0.322	0.3 (-0.5;1.2)	0.401
Osteocalcin (ng/mL)	-0.0 (-0.0;0.0)	0.230	-0.0 (-0.0;0.0)	0.114	0.0 (-0.0;0.0)	0.189	0.0 (-0.0;0.0)	0.139	-0.0 (-0.0;0.0)	0.079	-0.0 (-0.0;0.0)	0.364
R^2^ ^4^	**0.13**		**0.16**		**0.12**		**0.15**		**0.08**		**0.11**	
**BMD - Femoral neck (FN)**												
FN (g/cm^2^)	-0.1 (-0.6;0.3)	0.553	-0.5 (-1.0;0.0)	0.052	0.2 (-0.2;0.6)	0.385	0.5 (-0.0;1.0)	0.066	-0.3 (-0.6;-0.0)	**0.038**	-0.2 (-0.5;0.1)	0.245
WC (normal/increased)^3^	-0.4 (-1.3;0.5)	0.395	-0.3 (-1.3;0.5)	0.440	0.4 (-0.5;1.3)	0.383	0.3 (-0.5;1.2)	0.457	-0.2 (-0.8;0.3)	0.392	-0.1 (-0.7;0.4)	0.606
*Interaction term*												
FN#WC	1.0 (0.1;1.9)	**0.032**	1.0 (0.1;1.9)	**0.036**	-1.0 (-1.9;-0.1)	**0.033**	-0.9 (-1.8;-0.0)	**0.042**	0.6 (-0.0;1.2)	**0.061**	0.4 (-0.1;1.0)	0.135
Osteocalcin (ng/mL)	-0.0 (-0.0;0.0)	0.269	-0.0 (-0.0;0.0)	0.119	0.0 (-0.0;0.0)	0.228	0.0 (-0.0;0.0)	0.144	-0.0 (-0.0;0.0)	0.110	-0.0 (-0.0;0.0)	0.378
R^2^ ^4^	**0.13**		**0.17**		**0.13**		**0.16**		**0.09**		**0.11**	
**BMD - Proximal femur (PF)**												
PF (g/cm^2^)	-0.1 (-0.5;0.4)	0.799	-0.5 (-1.0;0.0)	0.080	0.1 (-0.3;0.5)	0.571	0.4 (-0.1;0.9)	0.098	-0.3 (-0.6;-0.0)	0.047	-0.2 (-0.5;0.2)	0.307
WC (normal/increased)^3^	-0.2 (-1.2;0.7)	0.617	-0.2 (-1.2;0.7)	0.638	0.2 (-0.7;1.2)	0.605	0.2 (-0.7;1.1)	0.647	-0.2 (-0.8;0.4)	0.591	-0.1 (-0.7;0.5)	0.726
*Interaction term*												
PF#WC	0.8 (-0.1;1.7)	**0.090**	0.8 (-0.1;1.7)	**0.086**	-0.8 (-1.6; 0.1)	**0.091**	-0.7 (-1.6;0.1)	**0.097**	0.4 (-0.1;1.0)	0.137	0.4 (-0.2;1.0)	0.205
Osteocalcin (ng/mL)	-0.0 (-0.0; 0.0)	0.248	-0.0 (-0.0;0.0)	0.113	0.0 (-0.0;0.0)	0.212	0.0 (-0.0;0.0)	0.138	-0.0 (-0.0;0.0)	0.106	-0.0 (-0.0;0.0)	0.373
R^2^ ^4^	**0.13**		**0.16**		**0.12**		**0.15**		**0.08**		**0.11**	

1 Linear regression coefficient;

2 p-value refers to linear regression.

3 Categorized using World Health Organization cutoff points, normal WC: < 80 cm for women and < 94 cm for men; increased WC: ≥ 80 cm for women and ≥ 94 cm for men.

4 R^2^ refers to the outcome variation explained by the models.

Model 1 was adjusted only for osteocalcin and the interaction between BMD and WC, Model 2 was further adjusted for physical activity, smoking, alcohol intake, sex, age and skin color. All the multiple models were statically significant (p-value < 0.001).

CI: confidence interval; HOMA: homeostatic model assessment; HOMA1-IR: insulin resistance; HOMA2-S: insulin sensitivity; HOMA2-β: β-cell function (insulin secretion); WC: waist circumference; BMD: bone mineral density.

## DISCUSSION

The present study has three main results. First, we found that BMD predict alterations in glucose metabolism in young adults. Second, we observed that the direction of the association differed according to WC classification, i.e., adults with increased WC had a positive association between BMD and IR, while those with normal WC had an inverse association between these two markers. The association between BMD and insulin sensitivity occurred in the opposite direction, i.e., we observed an inverse association in individuals with increased WC and a positive association in those with normal WC. Finally, we did not observe any significant association between OC and glucose metabolism in the adjusted models.

This study has some potential limitations. Although we used a large sample size from a birth cohort, only 24.9% (n = 513/2,063) of the individuals evaluated in the fourth phase of the birth cohort follow-up consented to undergo DXA assessments, and after exclusions, the final sample comprised 468 subjects who had valid BMD measurements. In addition, although WC is a very practical and internationally used tool to evaluate the deposition of intra-abdominal fat, recommended by WHO (20), its use has as a limitation the fact that it does not separate visceral adipose tissue of the subcutaneous tissue. Moreover, it was not possible to use the WHO protocol to measure waist circumference (WC) in our study, because data collection occurred from 2002 to 2004, while the WHO STEPS protocol was published in 2008 (23). Additionally, this study was based on a cross-sectional analysis, a study design that cannot determine whether the results are merely associations or if BMD exerts a causal effect on glucose metabolism in these young adults. Finally, although in experimental studies OC uncarboxilated has been reported to be the metabolically active form (9,13), we did not differentiate plasma OC by gamma-carboxylation status, and our assessment included all forms of OC. The strength of this study is the number of young adults evaluated by DXA, a very accurate procedure for measuring bone density. Moreover, in the multivariate analysis, we evaluated the inclusion of co-variables based on a DAG that allows for the minimization of bias in epidemiological studies. DAGs allows the identification of the minimum sufficient adjustment to estimate the total and direct effect of a certain exposure on the studied outcome (21). To the best of our knowledge, this is only the second study that has evaluated if BMD, assessed by a gold standard measure (DXA), predict alterations in glucose metabolism (IR, sensitivity and secretion) in young adults.

This study provides new information about the association between bone and glucose metabolism. We found a significant association between BMD and IR and insulin sensitivity and a significant interaction between BMD and WC. A non-significant association between BMD and glucose metabolism (plasma glucose and serum insulin) has been found in the unadjusted model, which persisted after adjusting the analysis in 155 healthy young adults (24). In that study, although fat mass was considered a confounder, the adipose tissue was not tested as an effect modifier in the association between bone and glucose metabolism (24), as done in our study.

It is known that body fat, particularly visceral fat, may affect bone metabolism markers and BMD. Chronic low-grade inflammation associated with visceral fat is related to the genesis of osteoclasts, increased bone resorption and decreased OC concentration (11,12). Individuals with obesity present increased risk of fractures possibly associated with metabolic dysfunction that result in reduction of bone turnover and bone quality (25). Therefore, considering the effects of inflammation on bone turnover and mass, our conceptual framework considers that WC plays an important role in the association between BMD and IR. We have hypothesized that WC acts as an effect modifier and not as a confounder, and for this reason, this marker of visceral fat deposition was not included in the DAG that depicted the theoretical relationship between all involved variables.

OC is one of the most studied bone biomarkers in the association with glucose metabolism. In the current study, it was observed an inverse correlation between OC and WC in a sample of predominantly white young adults of both sexes (data not shown). These results corroborates with the inverse relationship between OC and visceral fat area found in Chinese men (12) and an inverse relationship between OC and trunk fat in men with obesity (26). These findings suggest a negative effect of adipose tissue, especially visceral fat, on OC.

Despite this, we did not find a significant association between OC concentrations and HOMA1-IR or HOMA2-S in either the crude or adjusted analysis. Animal studies, however, have demonstrated the positive effect of OC on insulin secretion and the sensitivity and proliferation of pancreatic β-cells (9,27). To exert these effects, OC binds to its receptor GPCR6a in pancreatic β-cells and can also increase the expression of anti-inflammatory adipokines and reduce the secretion of pro-inflammatory cytokines (13). In humans, the findings remain controversial. In line with our results, 137 young adults (18.6 years) were evaluated and no association was found between OC and HOMA1-IR (28). In addition, other studies found no association between OC and HOMA1-IR, HOMA2-β, QUICKI insulin sensitivity marker, blood glucose and insulin in pre- and post-menopausal women (14,29). On the other hand, some studies have found an inverse association between OC and IR and a positive association between OC and insulin sensitivity and secretion (12,26,30). The differences between these studies and ours may be explained by the fact that we studied healthy young adults while the others studies investigated older people (approximately 50 years of age) and/or individuals with obesity, which tend to have higher IR. Moreover, we also found methodological differences as most studies used only correlation statistical procedures (12,26), and only one study performed adjusted regression models like ours (30). Unlike our study, none of the published articles evaluated the selection of covariates with a DAG model. Finally, we expected that the addition of OC in the regression model could explain the association between BMD and IR, however, we observed that associations between bone and glucose metabolism is independent of this bone metabolism marker (because the inclusion of OC in the regression model did not affect the association between BMD and glucose metabolism).

Some studies have demonstrated that osteoprotegerin (OPG), that promotes bone formation, appears to be increased in metabolic disorders, such as obesity (31), and in individuals with obesity was found a positive association between OPG and HOMA1-IR (32). In experimental study, OPG increased inflammation in adipose tissue (33). This association of OPG with inflammation may explain its association with IR. In addition to OPG, the amino terminal propeptide of procollagen type 1 (P1NP), a marker of bone formation as OC, was also positively associated with HOMA1-IR in young women with overweight or obesity (15). In view of this, we suggest that further studies be performed to investigate the action of biomarkers other than OC, that may explain the positive association between BMD and HOMA1-IR observed in our study.

Individuals in the accrual phase present higher speed of bone mass gain, especially until reaching peak bone mass. Considering that in our study we found a positive effect of BMD on IR in young individuals with increased WC, it can be concluded that this is a critical phase of life, associated with increased metabolic risk. It is recognized that IR is involved in the pathophysiology of DM2, a global public health problem. The association between bone and IR suggests the existence of bone-pancreas axis. However, the exact mechanism that links bone mass and glucose metabolism is not fully understood, and this study sought to contribute evidence to clarify this relation. We believe that a better understanding of this association can contribute to improve IR. Corroborating this statement, other studies have been developed with the aim of modulating pharmacologically bone metabolism markers to improve glycemic control (34). Additionally, disorders associated with IR, such as obesity, seem related to reduced bone quality and formation and increased bone fracture risk (25). Thus, the investigation of the relationship between bone and glucose metabolism may not only contribute to the glycemic control but also to bone fragility prevention.

Moreover, as expected, we found that subjects with obesity and those with increased WC present higher IR and secretion and lower insulin sensitivity. Individuals with obesity were evaluated and it was identified that those with a higher percentage of lean mass also had higher insulin sensitivity and lower inflammatory status (35). Greater insulin secretion was found in individuals who have greater IR, which characterizes the pancreatic response in compensation of IR (1).

It is known that the increased secretion of adiponectin and the positive effect of estrogen on glucose homeostasis contributes to the lower IR observed in women compared to men (6), as found in our results. In addition, we found that men had a higher mean BMI compared to women, which may also explain the higher rate of IR in this group.

We conclude that BMD was associated with glucose metabolism and this association is independent of OC. We also found that the WC modifies the association between BMD and IR and sensitivity. These results indicate that bone may play a role in the metabolic profile of IR and obesity. However, further studies are needed to assess the direction of the association between BMD and IR and to test the possible mechanisms involved in this relationship.
